# Annual Report on Meteorology and Epidemics

**Published:** 1846-06-01

**Authors:** 


*Annual Report on Meteorology and Epidemics by* DR. MOORE.

This relates to an important province of medical fact and inquiry, and the reporter appears to have availed himself of all accessible particulars for the investigation. The interest of the paper is, however, chiefly local, and would not justify an analysis for this journal.

We again take occasion to express the gratification which these "transactions" afford, of the prosperity and usefulness of the College of Physicians at Philadelphia. It would be well for the Profession, if similar institutions existed in different parts of the country, manifesting similar zeal for scientific objects.

